# New Method of Early RRMS Diagnosis Using OCT-Assessed Structural Retinal Data and Explainable Artificial Intelligence

**DOI:** 10.1167/tvst.14.2.14

**Published:** 2025-02-10

**Authors:** Miguel Ortiz, Ana Pueyo, Francisco J. Dongil, Luciano Boquete, Eva M. Sánchez-Morla, Rafael Barea, Juan M. Miguel-Jimenez, Almudena López-Dorado, Elisa Vilades, María J. Rodrigo, Beatriz Cordon, Elena Garcia-Martin

**Affiliations:** 1 School of Physics, University of Melbourne, Victoria, Australia; 2 Department of Ophthalmology, Miguel Servet University Hospital, Zaragoza, Spain; 3 Aragon Institute for Health Research (IIS Aragon), Miguel Servet Ophthalmology Innovation and Research Group (GIMSO), University of Zaragoza, Zaragoza, Spain; 4 Biomedical Engineering Group, Department of Electronics, University of Alcalá, Alcalá de Henares, Spain; 5 Institute of Psychiatry and Mental Health, Gregorio Marañón University Hospital, IiSGM, Madrid, Spain; 6 Faculty of Medicine, Complutense University of Madrid, Madrid, Spain; 7 CIBERSAM: Biomedical Research Networking Centre in Mental Health, Madrid, Spain

**Keywords:** optical coherence tomography (OCT), relapsing-remitting multiple sclerosis (RRMS), multiple sclerosis (MS), early diagnosis, recursive feature extraction, Shapley additive explanations (SHAP), posterior pole protocol

## Abstract

**Purpose:**

The purpose of this study was to provide the development of a method to classify optical coherence tomography (OCT)-assessed retinal data in the context of automatic diagnosis of early-stage multiple sclerosis (MS) with decision explanation.

**Methods:**

The database used contains recordings from 79 patients with recently diagnosed relapsing-remitting multiple sclerosis (RRMS) and no history of optic neuritis and from 69 age-matched healthy control subjects. Analysis was performed on the thicknesses (average right and left eye value and inter-eye difference) of the macular retinal nerve fiber layer (mRNFL), macular ganglion cell layer (mGCL), macular inner plexiform layer (mIPL), and macular inner retinal complex layer (mIRL), dividing the macular area into six analysis zones. Recursive feature extraction (RFE) and Shapley additive explanations (SHAP) are combined to rank relevant features and select the subset that maximizes classifier (support vector machine [SVM]) performance.

**Results:**

Of the initial 48 features, 20 were identified as maximizing classifier accuracy (*n* = 0.9257). The SHAP values indicate that average thickness has greater relevance than inter-eye difference, that the mGCL and mRNFL are the most influential structures, and that the peripheral papillomacular bundle and the supero-temporal quadrant are the zones most affected.

**Conclusions:**

This approach improves the success rate of automatic diagnosis of early-stage RRMS and enhances clinical decision making transparency.

**Translational Relevance:**

Retinal structure assessment using OCT data could constitute a noninvasive means of diagnosing early-stage MS. This new high-accuracy and high-explainability method of analysis can be implemented in most hospitals and healthcare centers.

## Introduction

Alterations in the retinal layer structure in patients with multiple sclerosis (MS) were assessed using histological analysis^1^ and, noninvasively, using retinal optical coherence tomography (OCT) and angiography OCT (angio-OCT).^2^ Systematic literature reviews and meta-analyses demonstrate that OCT detects thinning of the retinal nerve fiber layer (RNFL), the ganglion cell layer (GCL), and the inner retinal complex layer (IPL), even in the early stages of MS.^3^^–^^5^

The current version of the McDonald criteria^6^ does not include use of retinal OCT scans to confirm dissemination in space, even though OCT procedures are used in MS diagnosis in 38% of cases worldwide.^7^ Recent research indicates the potential diagnostic advantages of adding OCT assessment of the optic nerve to current diagnostic criteria as a fifth analysis region.^8^^,^^9^

Previous papers have explored implementation of assisted MS diagnosis systems supported solely by artificial intelligence analysis of OCT recordings in the early stages of the disease.^10^^–^^13^

A key characteristic of OCT recordings is the high number of features they can obtain: depending on the acquisition equipment, it is possible to segment between 6 and 10 retinal structures. Moreover, for each structure, the technology can obtain information on either 40 × 60 points (Triton swept-source OCT) or 8 × 8 points (Spectralis OCT Fourier domain technology), among other options. Although literature review initially identifies the structures and zones most affected, it is of key importance to perform feature selection (FS) to optimize automatic classifier performance.

FS consists of selecting a subset of the initial features that maximizes the performance of a classifier. Recursive feature elimination (RFE) recursively discards the least relevant features. The initial features are trained and a ranking value is obtained based on their contribution to the classification. The least relevant features are then discarded and the procedure is repeated with the remaining ones until an optimal result is achieved. The first version of RFE, used to perform gene selection in cancer diagnosis, used a support vector machine (SVM) with a linear kernel^14^ as a classifier because it is possible to ascertain the relevance of each feature using the support vectors of this classifier.

Applied to diagnosis of early-stage relapsing-remitting multiple sclerosis (RRMS),^13^ it investigates the use of RFE with a linear-kernel SVM classifier to obtain the seven most relevant OCT features (3 relate to average thickness and 4 to inter-eye difference) and confirms the correlation with the clinical status of the patients as assessed on the Expanded Disability Status Scale (EDSS). The classifier based on the top-ranked features achieved an accuracy of 0.88.

Another feature-ranking option is to use Shapley additive explanations (SHAP) to assign a relevance value for a particular prediction to each feature,^15^ thus distinguishing between relevant features and irrelevant ones. The SHAP method is based on the Shapley game theory values used to explain the output of any individual model. In medicine, SHAP values have been used to select features in references.^16^^,^^17^

Given that there is room to improve the algorithms used in OCT-based diagnosis of early-stage RRMS, this paper aims to identify a subset of OCT features capable of effectively differentiating between healthy control subjects and patients. To enhance algorithm performance and generate insights into the input features’ contribution to the prediction, it proposes ranking the features using recursive feature elimination and SHAP values.

## Methods

This study was approved by the Clinical Research Ethics Committee of Aragon (Zaragoza, Spain) and conducted in accordance with the principles of the Declaration of Helsinki. All participants provided written informed consent after receiving a detailed explanation of the study. Data from these patients and controls were previously analyzed in references.^12^^,^^13^

The eligibility criteria for the patient cohort included the following: (1) RRMS phenotype and disease duration < 26 months, because the aim is to improve the diagnosis of early-stage RRMS (the most common MS phenotype); and (2) no history of optic neuritis (ON) in either eye (to exclude easily diagnosed subjects). The healthy controls had no history of ocular or neurological disease and presented no signs or symptoms of them. For the study group, the criteria were ages 17 to –70 years and tolerance of high-quality OCT examination.

The exclusion criteria for both cohorts were as follows: (1) best-corrected visual acuity < 0.5 (Snellen charts); (2) refractive errors > 5 diopters of spherical equivalent refraction or 3 diopters of astigmatism; (3) intraocular pressure > 20 millimeters of mercury (mm Hg); (4) media opacification (nuclear color/opalescence or cortical or posterior subcapsular lens opacity < 1 according to the Lens Opacities Classification System III) to exclude dense cataracts that can affect OCT thickness measurements (not common in patients with the mean age of this cohort); (5) concomitant ocular disease (including glaucoma or retinal pathology); and (6) other systemic conditions potentially affecting the visual system.

### OCT Method

The OCT recordings were made as per the APOSTEL recommendations.^18^ OCT measurements of both eyes were taken with a spectral-domain (SD) OCT (Spectralis, Heidelberg Engineering, Heidelberg, Germany) device using the posterior pole asymmetry analysis protocol.^19^ The protocol obtained the thicknesses of nine retinal structures. This study uses the four retinal structures that exhibit the greatest capacity to discriminate between control eyes and those of patients with RRMS: mRNFL, mGCL, mIPL, and macular inner retinal layer (mIRL) complex (mIRL = mRNFL + mGCL + mIPL).

The 25 × 30 explored area centered on the foveola was represented as an 8 × 8 grid that reflects overall retinal thickness. Each layer was segmented using the inbuilt software (HRA version 6.0.7.0). Scan quality was analyzed according to the OSCAR-IB Consensus Criteria for Retinal Quality Assessment.^20^ No manual correction was applied to the OCT output and only high-quality scans (25/40) were used for analysis.

**Figure 1.**
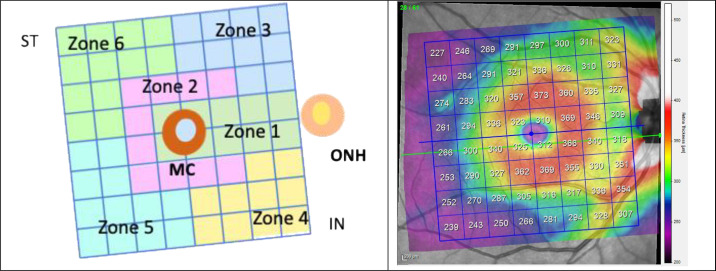
*Left*: Definition of the analysis zones on the posterior pole grid of the macular area of the right eye using the Spectralis optic coherence tomography (OCT) protocol. The posterior pole analysis protocol uses the Anatomic Positioning System (APS) to adjust the scans of the axis fovea to the center of Bruch's membrane. *Right*: Spectralis OCT recording showing macular thicknesses on the 8 × 8 grid that corresponds to the analysis zones defined in the figure on the *left*. ONH, optic nerve head; MC, macula center (fovea); IN, inferior nasal; ST, superior temporal.

The 8 × 8 grid was divided into 6 analysis zones: zone 1 = the central papillomacular bundle, zone 2 = the peripheral papillomacular bundle, zone 3 = the supero-nasal quadrant, zone 4 = the infero-nasal quadrant, zone 5 = the infero-temporal quadrant, and zone 6 = the supero-temporal quadrant (see Fig. 1).

The average thickness values (AVG) of the two eyes of each subject and the difference in thickness between the right eye and the left eye (DIFF) were both analyzed. The initial number of features per subject was as follows: 6 zones × 2 measurements × 4 structures = 48 features.

### Feature Selection and Classification

**Figure 2.**
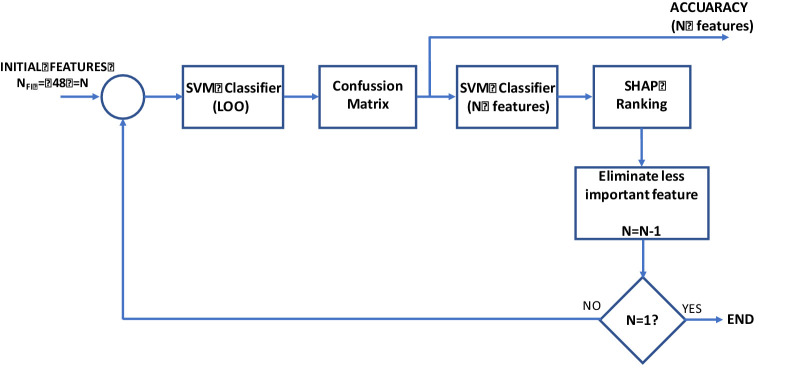
Diagram of the RFE–SHAP method. LOO, leave-one-out; SVM, support vector machine.

Automatic classifier performance deteriorates if the classifier uses a large number of inputs that do not contribute relevant information and may be correlated. There is no single broad solution to the feature selection problem. In this paper, RFE is used to create a SHAP ranking that assigns each feature a relevance value for a particular prediction^15^ (Fig. 2).

The details of this procedure are as follows:

a) The initial number of features is N_RFE_ = 48 = N.
b) The classifier is trained using this number of features and its confusion matrix is obtained using the leave-one-out (LOO) method and the degree of accuracy. The SHAP values, which assign a relevance value for this prediction to each input, are obtained.
c) The least relevant feature (N = N -1) is discarded and the process is repeated from step b onward until N = 1.

This process can be plotted (accuracy versus N) and the subset of features (N_RELEVANT_) that obtains the greatest accuracy value is selected.

A linear-kernel SVM classifier (C = 1.0 [regularization parameter], penalty = L2 and loss function = squared hinge) is used.

## Results

### Study Cohort and OCT Protocol

Table 1 shows the demographic and clinical characteristics of the two cohorts. The RRMS group comprises 68 female subjects and 11 male subjects, whereas the healthy group comprises 46 female subjects and 23 male subjects.

**Table 1.** Demographic and Clinical Characteristics of the Patients With Multiple Sclerosis and Healthy Controls

	RRMS Patients (*n* = 79 Patients)	Healthy Controls (*n* = 69 Subjects)	*P* Value
Age, y, mean ± SD	45.64 ± 13.59	46.94 ± 12.64	0.604
Visual acuity, Snellen	0.83 ± 0.57	0.91 ± 0.36	0.026
Disease duration since definitive diagnosis, y, mean ± SD	1.42 ± 0.72	—	—
EDSS score, median (range)	1.28 (1–3)	—	—
Treatment	Avonex: 3	—	—
	Betaseron: 7		
	Rebif: 6		
	Capaxone: 6		
	Tecfidera: 11		
	Gilenya: 32		
	Mayzent: 3		
	Aubagio: 9		
	No treatment: 2		

EDSS, Expanded Disability Status Scale; RRMS, relapsing-remitting multiple sclerosis; SD, standard deviation.

**Figure 3.**
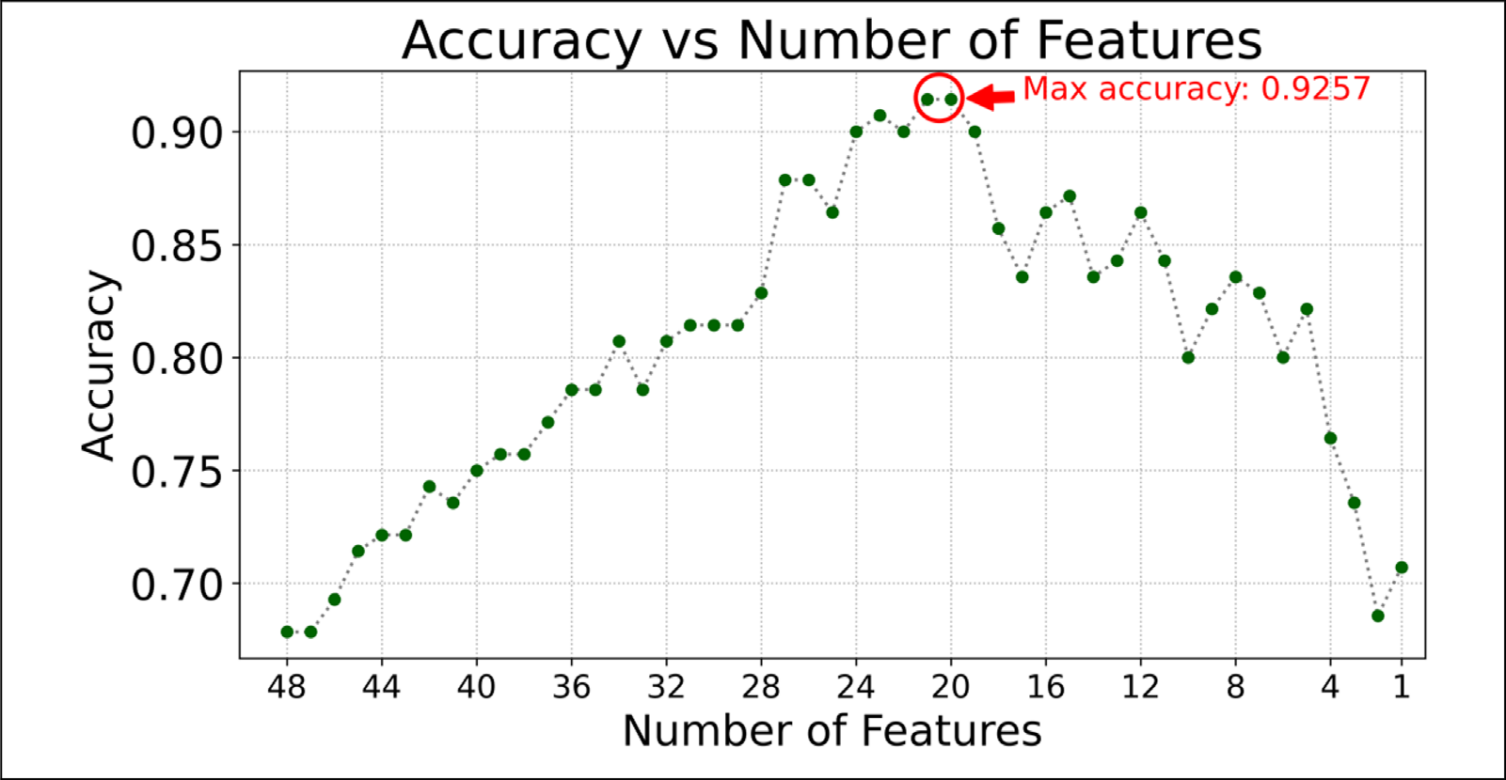
RFE–SHAP algorithm performance.

Figure 3 shows the results obtained using the RFE–SHAP method. Model performance peaked (accuracy = 0.9257) at 21 and 20 features. The analysis below is performed with the N_RELEVANT_ = 20 features that make the most significant contribution to the classification.

**Figure 4.**
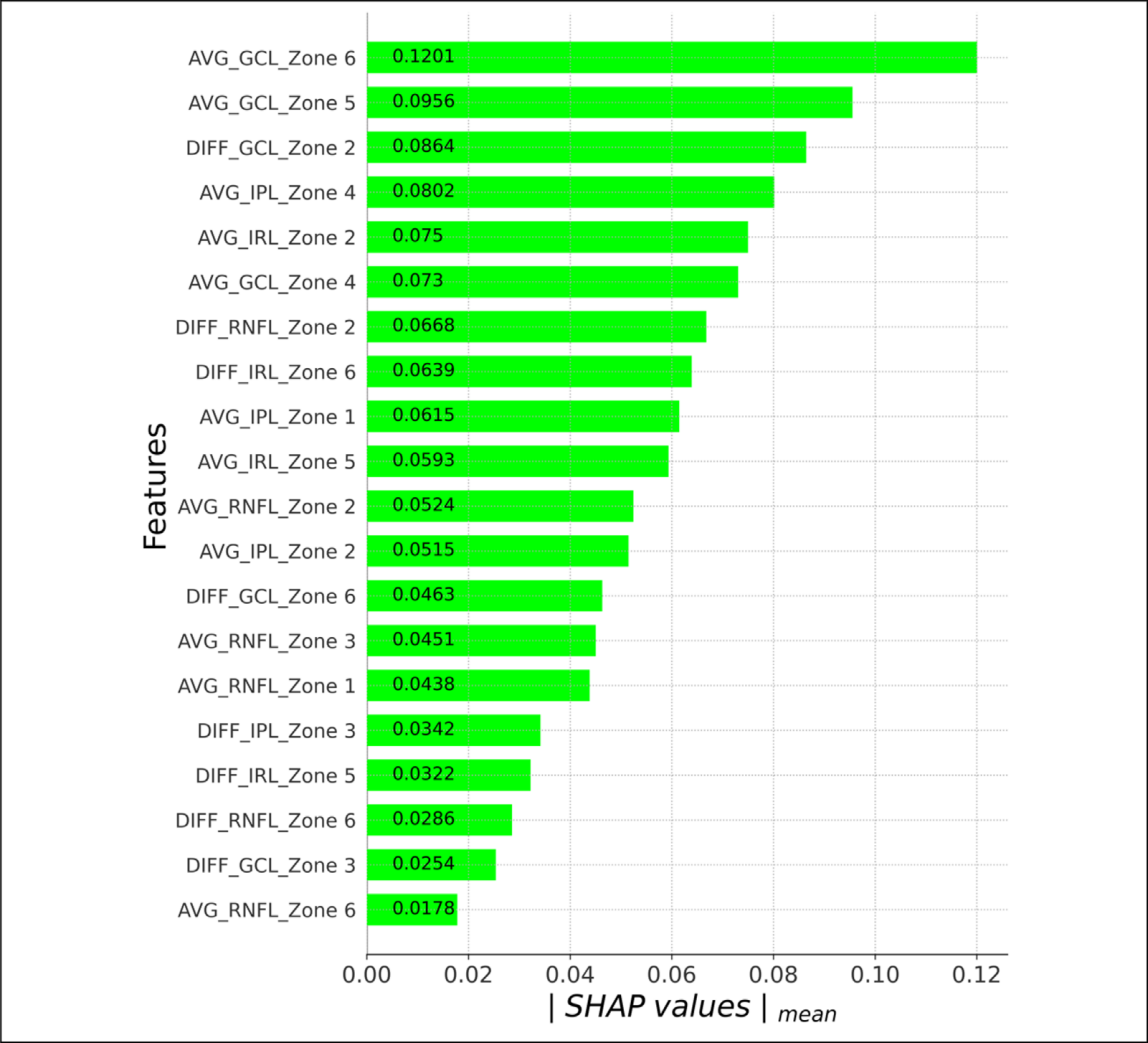
Ranking of relevance of the selected features as per absolute mean SHAP value.

Figure 4 shows the ranked relevance of each classifier input variable. The SHAP values vary throughout the range (0.1201–0.0178) and decrease progressively, meaning that a threshold value that identifies a group of features that are clearly more relevant than others cannot be determined. Of the 20 features that influence the classifier, 12 correspond to average thickness values (aggregate SHAP values = 0.7753) and 8 to the inter-eye difference (aggregate value = 0.3838). In addition, seven entries in the top half of the ranking are average thickness values and three are inter-eye difference values. Therefore, average thickness has a greater influence on the classification than inter-eye difference.

In Figure 4, the retinal structures most commonly identified are the GCL (n_GCL_ = 6; the top 3 values correspond to this layer and the aggregate value for the entire GCL is 0.4468), the RNFL (n_RNFL_ = 6; aggregate value = 0.2545), the IPL (n_IPL_ = 4; aggregate value = 0.2274), and the IRL (n_IRL_ = 4; aggregate value = 0.2304). Consequently, the structures that most condition the classifier decision are, in order of relevance, the GCL, RNFL, IPL, and IRL.

The results of the SHAP ranking for the zones are as follows: zone 2 (n_ZONE2_ = 5; aggregate value = 0.3321), zone 6 (n_ZONE6_ = 5; aggregate value = 0.2767), zone 5 (n_ZONE5_ = 3; aggregate value = 0.1871), zone 4 (n_ZONE4_ = 2 aggregate value = 0.1532), zone 1 (n_ZONE1_ = 2; aggregate value = 0.1053), and zone 3 (n_ZONE3_ = 3; aggregate value = 0.1047). It can be deduced that the zones that most influence the classifier are zone 2 (peripheral papillomacular bundle) and zone 6 (supero-temporal quadrant).

Training the classifier with the 20 features of interest and using the LOO validation method obtains the metrics presented in Table 2.

**Table 2.** Classifier Metrics for the 20 Most Relevant Features

Metric	Value
Accuracy	0.9257
Sensitivity	0.9747
Specificity	0.8696
F1-score	0.9333

## Discussion

This paper implements a recursive OCT feature selection method based on SHAP ranking that, using only retinal structure data, enhances early RRMS diagnosis and also explains the decisions made by the classifier.

The main outcome of this approach is that it improves on prior methods of automatic RRMS diagnosis^12^^,^^13^ and provides insights into how RRMS affects the neuroretina in the initial stages of the disease in patients without ON.

A previous approach that applied a convolutional neural network to the same database obtained an accuracy value of 0.87^12^ but did not perform feature selection or analyze the explainability of the results.

The classic RFE–SVM method identifies 7 relevant variables and achieves an accuracy of 0.88.^13^ In contrast, RFE–SHAP achieves an accuracy of 0.9257 and identifies 20 relevant features. The difference in the results produced by the two RFE methods can be explained by the fact that they are built on different mathematical foundations: in the first case, relevance is assessed using support vector distance, whereas, in the second method, assessment is based on the SHAP value, which analyzes the marginal value each feature contributes to the classifier output.

Most studies using OCT to analyze retinal structure in early-stage RRMS provide evidence of the effect on the average thickness of certain retinal structures, including in patients with no history of ON.^12^^,^^13^^,^^21^ Inter-eye difference has recently gained importance in RRMS diagnosis in both advanced^22^^,^^23^ and early stages of the disease.^12^^,^^13^

Among the 20 features considered relevant, greatest relevance is assigned to values that assess the average of the left and right eye thicknesses in each subject. It is also evident that inter-eye difference contributes to a lesser degree to differentiation between patients with RRMS.

This paper analyses data from the four structures that have the greatest capacity to differentiate patients with RRMS evaluated using the area under the curve (AUC): mRNFL, mGCL, mIPL, and mIRL. Our findings are in line with those obtained in reference ^24^, which analyzed patients with RRMS without ON and with a disease duration of 23 ± 13 months, observing that mRNFL, mGCL, mIPL, and mGCIPL thickness (the authors did not analyze IRL) were significantly reduced. Martucci et al.^25^ use the posterior pole protocol to analyze patients (97 eyes without ON and a disease duration of 43.2 ± 66.7 months versus 106 control eyes). The findings indicate that there is a significant difference in GCL thickness (*P* < 0.001) and, although it is not statistically significant, the INL (*P* = 0.072) and RNFL (*P* = 0.074) are thinner.

Most previous papers observe that the greatest discriminant capacity is found in the GCL or GCIPL complex (GCL+IPL), generally followed by the RNFL. The findings of this study support this observation because, according to the SHAP values, the GCL plays a greater role in classifier decision making than the RNFL. This may be due to the fact that significant thinning of the GCL appears in the first month from symptom onset, whereas RNFL thinning is not detected until at least 3 months after symptom onset.^26^ GCL is made up of ganglion cell somas, which decrease in size in neurodegenerative pathologies due to the anterograde atrophy caused by the axonal damage produced in the central nervous system (third neuron of the visual pathway) and which advances to the second neuron of the visual pathway (which is the ganglion cell; the first neuron is the retinal bipolar cell). Another complementary explanation may be that segmentation of the RNFL often depends on certain ocular conditions (e.g. edema or inflammation), which makes the values in this layer less consistent when compared with the greater reproducibility of the GCL measurements.

According to the feature ranking obtained using the SHAP method, the areas with greatest influence on the classifier are the peripheral papillomacular bundle and the supero-temporal quadrant. Few papers identify defect patterns in retina structures in patients with early-stage RRMS and no history of ON. There are several ways of identifying zonal differences between patients and control subjects: statistical methods, Cohen distance, AUC, the SHAP method, etc.

The study by Albano et al. analyzes 34 eyes of patients with RRMS without ON (disease duration: 0.3 [0.05–1.98] years) and 34 control eyes and uses a statistical method to identify defects in the inner retinal layer complex (macular ganglion cell–inner plexiform layer complex).^27^ In line with our findings, the study reports that the most frequently occurring defect presents in the proximity of the peripheral papillomacular bundle.

Meanwhile, using the Cohen distance and SD-OCT recordings, Garcia-Martin et al.^10^ and López-Dorado et al.^11^ detect clear thinning of the perimacular area. This thinning principally affects the papillomacular bundle in the GCL+ (GCL+IPL) and GCL++ (RNFL+GCL+IPL) whereas little alteration is detected in the RNFL. Ortiz et al. uses the area under the receiver operating characteristic curve (AUROC) and observes that the defects mainly present in the GCL, IPL, and IRL, with the greatest thinning occurring in the perimacular areas.^12^

Also in line with our findings, using RFE with a random forest classifier identifies a set of eight retinal features in children (<18 years old) with MS (disease duration: 0.6 ± 1.5 years).^28^ An accuracy value of 0.80 is obtained in diagnosis of MS versus control subjects and it is observed that ganglion cell–inner plexiform layer thickness in the supero-temporal sector and retinal nerve fiber layer thickness in the temporal quadrant are among the features with greatest capacity to distinguish between patients with MS and healthy control subjects.

This study confirms the utility of the inner layers of the neuroretina in reliably diagnosing early-stage RRMS. It also serves to confirm the trend of previous studies in which artificial intelligence (AI) is used to optimize OCT-based diagnosis of this pathology. It likewise improves on previous outcomes and focuses on macular regions, which, according to the latest studies, are the ones that provide the most relevant information about this pathology. Furthermore, because this method relies less on inter-eye difference it may be more useful in subjects who have not presented ON in either eye, who are in the early stages of the disease, or in whom only one eye is assessable because the other eye has a history of trauma, anisometropia, lazy eye, etc.

A cross-sectional study with larger numbers of subjects in both groups would give the findings greater weight. In addition, because only patients with the relapsing-remitting phenotype were evaluated (as this is the most common phenotype of this disease), similar studies would be necessary for other MS phenotypes. Inclusion of other clinical characteristics in addition to ocular characteristics could further improve the performance of the algorithm. In future studies, incorporation of Angio OCT using new software designed by Heidelberg would provide a wealth of 3D information, including analysis of retinal irrigation and vascularization.

In conclusion, this approach improves both the success rate of automatic diagnosis and the transparency of the decision, thus enhancing the potential clinical utility and acceptance of OCT analysis in RRMS diagnosis. Building on previous papers^8^^,^^9^^,^^29^ it further contributes to the evidence supporting the inclusion of OCT-assessed optic nerve topography as a fifth analysis region capable of meeting the dissemination in space condition necessary when diagnosing MS according to the McDonald criteria.
